# Percutaneous iliosacral screw and trans-iliac trans-sacral screw with single C-arm fluoroscope intensifier is a safe treatment for pelvic ring injuries

**DOI:** 10.1038/s41598-021-04351-z

**Published:** 2022-01-10

**Authors:** Jui-Ping Chen, Ping-Jui Tsai, Chun-Yi Su, I.-Chuan Tseng, Ying-Chao Chou, I.-Jung Chen, Pai-Wei Lee, Yi-Hsun Yu

**Affiliations:** 1 Department of Orthopedic Surgery, Musculoskeletal Research Center, Chang Gung Memorial Hospital, No. 5, Fuxing St., Guishan Dist., Taoyuan City, 333423 Taiwan; 2 Department of Orthopedic Surgery, Keelung Chang Gung Memorial Hospital, No. 201, Maijin Rd., Anle Dist., Keelung City, 204011 Taiwan; 3 Department of Orthopedic Surgery, Taoyuan Chang Gung Memorial Hospital, No. 123, Dinghu Rd., Guishan Dist., Taoyuan City, 333008 Taiwan; 4 Center for Big Data Analytics and Statistics, Chang Gung Memorial Hospital, No. 5, Fuxing St., Guishan Dist., Taoyuan City, 333423 Taiwan

**Keywords:** Outcomes research, Risk factors, Trauma

## Abstract

To elucidate the accuracy, efficacy, and safety of percutaneous iliosacral screw (ISS) and trans-iliac trans-sacral screw (TITS) insertion using a single C-arm fluoroscopy intensifier. Additionally, the potential risk factors that might cause mal-positioned screws were identified. Patients with pelvic ring injuries who underwent percutaneous screw fixation in a single medical institute were divided into an ISS group (n = 59) and a TITS group (n = 62) and assessed. The angles deviated from ideal orientation (ADIO) of the implanted screw were measured, and potential risk factors for mal-positioned screws were analyzed. Overall, the reduction quality of the pelvic ring was good or excellent in 70 patients (82.4%) by Matta’s criteria and in 48 patients (56.5%) by Lefaivre’s criteria. ADIO measurements of the ISS and TITS groups via multi-planar computed tomography were 9.16° ± 6.97° and 3.09° ± 2.8° in the axial view, respectively, and 5.92° ± 3.65° and 2.10° ± 2.01° in the coronal view, respectively. Univariate statistical analysis revealed body mass index as the single potential risk factor of mal-positioned screws. With careful preoperative planning and intraoperative preparations, placing ISS and TITS under the guidance of single C-arm fluoroscopy intensifier is a reliable and safe technique. Caution should be exercised when performing this procedure in patients with a high body mass index.

## Introduction

Pelvic fractures represent 5–9.3% of all traumatic fractures, and 10–20% of poly-traumatized patients have pelvic fractures^1–3^. The treatment of pelvic fracture can be challenging, owing to the complexity of fracture patterns, individual anatomical variations, and nearby vital structures^4^. As the posterior pelvic ring provides 60–70% of the stability of the pelvis, anatomical reduction with adequate fixation is crucial in cases with posterior pelvic ring injury^5^. Therefore, the operative strategy for pelvic ring injury should be individualized and optimized according to each patient’s clinical presentation, including options of surgical approach, choice of implants, and the operation setting.

Since restoring pelvic anatomy and providing sufficient stability of the posterior pelvic ring are the keystones of treatment of pelvic ring injury, anatomical reduction should be performed prior to osteosynthesis. After achieving reduction of the fracture, by either closed or open methods, there are several options to maintain the reduction, reduce the gap, and achieve stability^6–8^. Among the fixation choices, percutaneous screw fixation via either closed or open reduction is usually preferred because it is less invasive, has minimal blood loss, a shorter surgical time, and adequate stability^9–11^. Despite its advantages, several concerns persist regarding this percutaneous technique, including the disadvantages of frequent X-rays, great cumulative radiation exposure, high screw position error rate, and nerve injury^12–14^.

Percutaneous treatment for posterior pelvic ring was first described by Matta et al. in 1980s^15^. The original description of the method was conducted under real-time image examination by a single-arm fluoroscopic intensifier. Since that time, several real-time image modifications have been proposed to improve the accuracy and safety of this technique, such as 2-arm fluoroscopy, O-arm fluoroscopy, intraoperative computed tomography, and navigation system^16–19^. Although reports from these new image-assisted surgery have revealed their efficacies, the advanced image tools are expensive and not routinely available to each orthopedic surgeon and facility. Therefore, the usefulness of these advanced image tools for the percutaneous treatment of posterior pelvic ring may be limited.

The aim of this study was to report the surgical outcomes using the most commonly available intraoperative image evaluation, the single fluoroscopic intensifier, in treating posterior pelvic ring injuries by either iliosacral screw (ISS) or trans-iliac trans-sacral screw (TITS) insertion. Additionally, the risk factors of potentially mal-positioned screws were evaluated.

## Materials and methods

### Patient enrollment

We retrospectively collected patients’ medical records from the fracture registration database of our institute to identify those who were diagnosed with pelvic ring injury and underwent percutaneous screws fixation (ISS, TITS, or both) either through closed reduction and internal fixation or open reduction and internal fixation (ORIF) between January 2017 and June 2020. The medical records and pre- and postoperative radiological images were meticulously reviewed. All the operative procedures were performed with an established operative protocol by a single surgeon (Y.-H.Y.). The review process was approved by the Chang Gung medical foundation institutional review board (IRB No. 202100620B0).

### Resuscitation and perioperative protocol

The patients were sent to the emergency department (ED) directly from trauma scenes or transferred from primary medical institutes. Advanced Trauma Life Support^®^ resuscitation protocol was followed in the ED, and then patients were transferred to the ordinary ward or intensive care unit, as required. Osteosynthesis for the pelvic fracture was performed immediately after the patient was hemodynamically stabilized. Image examinations, including X-rays [anteroposterior (AP), inlet, and outlet views] and multi-planar computed tomography (mpCT), were required for preoperative planning. Subsequently, the rehabilitation protocol was individualized according to the patient’s concomitant injuries and fractures. Similar postoperative image examinations were performed to examine the reduction quality of the pelvic ring and the position of the implants.

### Operative technique

Patients were positioned in supine or prone position, according to the planned procedures and the concomitant injuries observed on a radiolucent table (Modular Table System; Mizuho OSI, California, USA) under general anesthesia. In patients for whom a prone position was contraindicated because of concomitant injuries or anesthesia requirements, a supine position was preferred. However, a prone position was necessary for ORIF of posterior pelvic ring injuries, such as dislocated sacroiliac joint and vertical displacement of the sacral fracture. The osteosynthesis strategy for anterior and posterior pelvic ring injury could be performed simultaneously or sequentially. Posterior pelvic ring reduction and fixation was always performed prior to anterior pelvic ring procedure, except in cases with pelvic ring injury to AO B2.1^20^ with significant internal rotational deformity of the affected hemipelvis.

AP, inlet, outlet, 2 Judet views, and sacrum lateral views were examined prior to surgical draping to ensure that all the images could be obtained clearly without limitations. The intraoperative images were obtained from a single-arm fluoroscopic intensifier (Ziehm Solo; Ziehm Imaging GmbH, Nuremberg, Germany). The ideal orientation of the ISS was perpendicular to the sacroiliac joint, whereas that for TITS was parallel to the groundline. After the position and orientation of the K-wire was confirmed, a 7.0-mm cannulated screw (Cannulated Screw 7.0 mm; Syntec Technology Co., Hsinchu, Taiwan) was applied as the target implant. All images were examined repeatedly throughout the procedures. Moreover, the radiation dose and time were recorded thoroughly during percutaneous screw osteosynthesis.

### Analysis of screw placement and reduction quality of the pelvis

Standard X-rays (AP, inlet, outlet views) and mpCT were obtained postoperatively for each patient. Radiological interpretations were performed by two independent medical doctors (J.-P.C. and P.-J.T.), who were not involved in the surgeries, using the PACS system (Centricity Enterprise Web V3.0; GE Healthcare, Chicago, USA).

The morphology variability of the sacrum, in terms of sacral dysmorphism, was examined prior to osteosynthesis. We adapted the criteria by Routt^21^ to define a dysmorphic sacrum which are the signs from X-rays, including (1) mammillary bodies; (2) tongue-in-groove morphology; (3) collinearity; (4) dysmorphic neural foramina; (5) residual sacral disc space. When the sacral dysmorphism was anticipated from preoperative image evaluation, percutaneous osteosynthesis by TITS would be applied more frequently than that by ISS because of the narrow corridor of S1.

Several classifications and grading systems for fracture pattern, reduction quality evaluation, and screw positions were adapted in this study. We classified the fracture pattern according to the Arbeitsgemeinschaft für Osteosynthesefragen (AO) system or pelvic ring injury^20^. For those fractures with sacral involvements, the Denis classification were applied^22^. The reduction qualities of the pelvic ring injuries were evaluated from the pelvic AP, inlet, and outlet X-rays, and axial and coronal views of the mpCT. We adapted the criteria from Matta and Tornetta for vertical reduction quality^15,23^ and from Lefaivre for symmetrical reduction quality^24^. Accordingly, we classified the reduction quality of the pelvic ring injury as excellent, good, fair, or poor.

**Figure 1:**
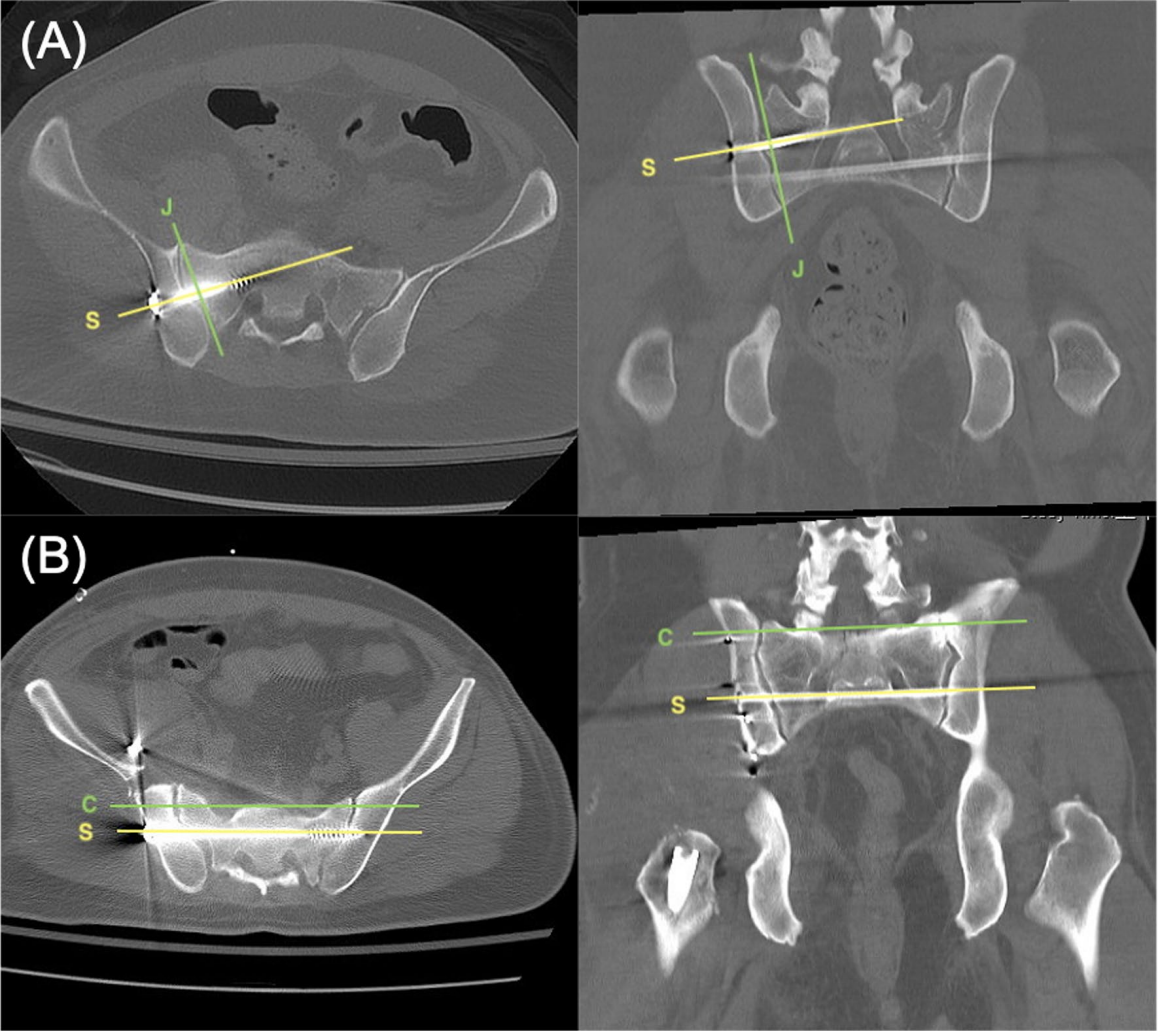
(**A**) The ideal angles between ISS (line S) and sacroiliac joint (line J) are 90° in both the axial and coronal views. The actual angles deviated from these ideal orientations are defined as the ADIO of ISS. (**B**) In both axial and coronal views, the ideal angle between TITS (line S) and groundline (line C) is 0°. The actual angles deviated from these ideal orientations are defined as the ADIO of TITS. *ISS* iliosacral screw, *TITS* trans-iliac trans-sacral screw, *ADIO* angle deviated from ideal orientation.

For ISS, we collected the angles between the screw and sacroiliac joint obtained in the axial and coronal views of the mpCT. The angular differences between the implanted screw and the ideal orientation of the ISS, which should be perpendicular to the sacroiliac joint in each view, were measured and termed as the angles deviated from ideal orientation (ADIO) (Fig. 1A). For TITS, the angular difference between the implanted screw and the ideal orientation, which should be parallel to the groundline in both axial and coronal views of the mpCT scan, were recorded (Fig. 1B).

To qualitatively define mal-positioned ISS and TITS, a screw that penetrated, encroached, or touched the neuroforamina of the sacrum or a newly appeared postoperative neurological deficit was defined as a mal-positioned screw, and Smith’s grading system (Table 1), which classified the position of the screw from grade 0 to 3, was applied to quantify those with mal-positions^25^.

**Table 1:** Definitions and results of mal-positioned iliosacral screw and trans-iliac trans-sacral screws as per Smith grading system.

	Grade 0	Grade 1	Grade 2	Grade 3
Perforation	No perforation	< 2 mm	2–4 mm	> 4 mm
Angulation	< 5°	5°–10°	11°–15°	> 15°

### Statistical analysis

The Chi-square test or the Fisher’s exact test was used where appropriate to analyze categorical data. Nonparametric Mann–Whitney U test was applied for between-group comparisons in numerical data. Logistic regression was applied for analysis of risk factors. Statistical significance was defined as p < 0.05. Statistical analysis was carried out by SPSS 24.0 program for Windows (IBM SPSS Statistics for Windows, Version 24.0; IBM Corp, NY, USA).

### Ethics approval and consent to participate

Informed consent was obtained from all subjects and approval from the Chang Gung medical foundation institutional review board (202100620B0) was acquired. All methods were performed in accordance with Declaration of Helsinki.

## Results

### Demographics

From January 2017 to June 2020, 85 patients with a mean age of 39 ± 19 were included in this study. We divided the enrolled patients into two groups: ISS and TITS. Among the 59 cases in the ISS group, 47 had an AO type B injury and 12 had a type C injury. Meanwhile, among the 62 patients in the TITS group, there was 1 with AO type A injury, 48 with type B injury, and 13 with type C injury. Thirty-six patients received both ISS and TITS treatments. Twenty-four patients had diastatic sacroiliac joints, 17 of whom received open reduction (70.8%). Eight patients were found to have sacral dysmorphism (incidence: 6.6%). The details of demographic data are listed in Table 2.

**Table 2:** Demographic data of the patients who underwent percutaneous iliosacral screw (ISS) and trans-iliac trans-sacral screw (TITS) fixation.

	ISS	TITS
Number	59	62
Age	36 (16–82)	41 (16–82)
**Sex**		
Male	34	31
Female	25	31
BMI	23.6 (15.1–39.2)	23.4 (15.1–39.2)
**Injury mechanism**		
Motorbike accident	30	29
Car accident	5	5
Fall from height	10	10
Crush	8	10
Others	6	8
Injury Severity Score	21.5 (2–48)	19.7 (4–48)
Open fracture	5 (8.5%)	6 (9.7%)
**AO classification**		
A	0	1
B	47	48
C	12	13
**Sacral fracture**^a^	26 (44.1%)	40 (64.5%)
Zone I	16	24
Zone II	10	16
Sacral dysmorphism	0	8 (12.9%)

*BMI* body mass index, *AO* Arbeitsgemeinschaft für Osteosynthesefragen.

^a^Denis classification.

### Surgical results

The reduction quality of the pelvic ring injury was classified as excellent or good in 70 patients (82.4%) by Matta’s criteria and in 48 patients (56.5%) by Lefaivre’s criteria. The ADIO measurements of the ISS and TITS groups were 9.16° ± 6.97° and 3.09° ± 2.8° in the axial view, respectively, and 5.92° ± 3.65° and 2.10° ± 2.01° in the coronal view, respectively (Table 3). The radiation dose in ISS and TITS groups were 40.67 ± 15.42 mGy and 42.55 ± 18.35 mGy, respectively. Further, radiation time was 118.23 ± 29.22 s for a single ISS, and 132.58 ± 38.19 s for a single TITS. Additionally, according to the definition by Smith et al.^24^, the evaluations of ISS in both views had grade 1 mal-angulation and grade 0 perforation, whereas those of TITS showed grade 0 mal-angulation and grade 0 perforation.

**Table 3:** Surgical results of the patients with percutaneous surgery for the pelvic ring injuries.

	ISS	TITS
**ADIO on mpCT**		
Axial view (°)	9.16 ± 6.97	3.09 ± 2.85
Coronal view (°)	5.92 ± 3.65	2.10 ± 2.01
**Evaluation of reduction quality of the pelvic ring injury** **Matta**		
Excellent	27 (45.8%)	31 (50.0%)
Good	22 (37.3%)	20 (32.3%)
Fair	10 (16.9%)	11 (17.7%)
Poor	0	0
**Lefaivre**		
Excellent	17 (28.8%)	18 (29.0%)
Good	15 (25.4%)	18 (29.0%)
Fair	19 (32.2%)	23 (37.1%)
Poor	8 (13.6%)	3 (4.8%)
Neuroforamen perforation	0	0

*ISS* iliosacral screw, *TITS* trans-iliac trans-sacral screw, *ADIO* angle deviated from ideal orientation, *mpCT* multi-planar computed tomography.

### Complications

One surgical site infection from the percutaneous wound was found 7 days after the index surgery. The wound finally healed uneventfully with implant retention after surgical debridement, proper wound care, and adequate systemic antibiotic treatment.

### Risk factors analyses

**Figure 2:**
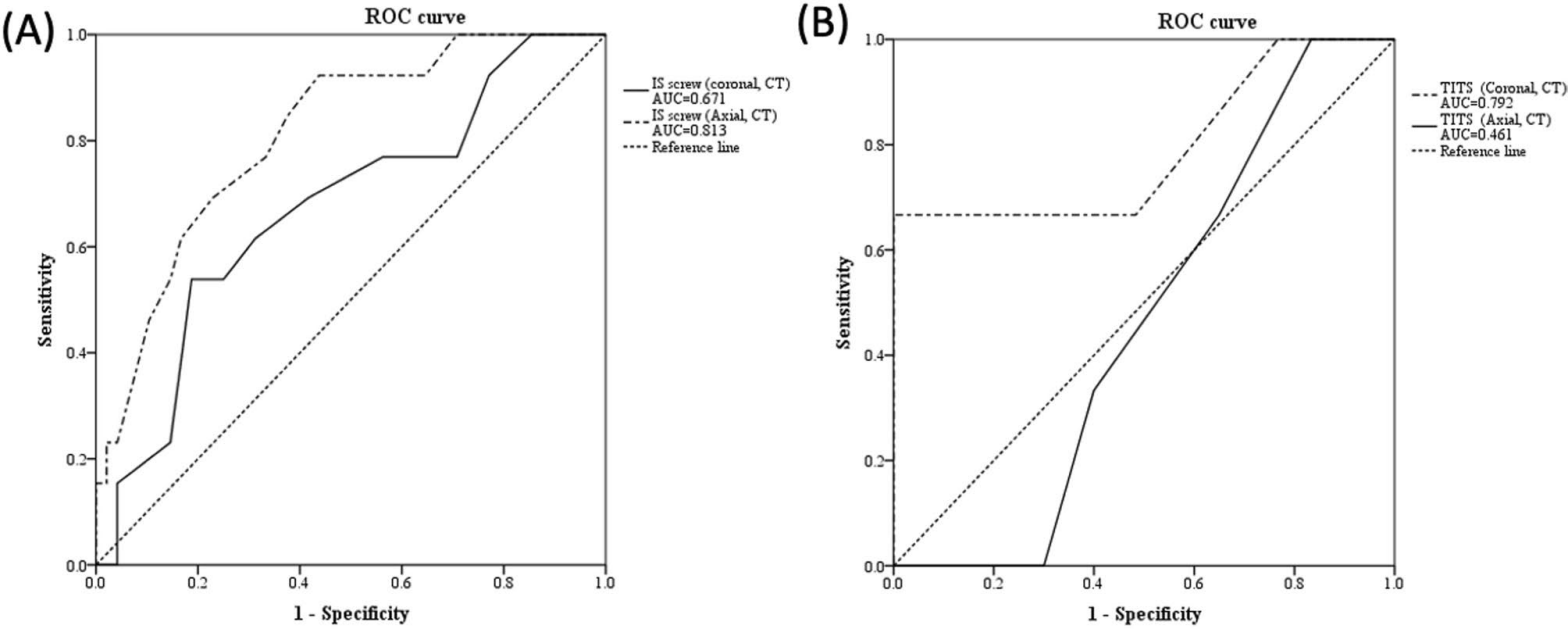
Receiver operating characteristic curve in (**A**) iliosacral screw group and (**B**) trans-iliac trans-sacral screw group.

To determine the potential risk factors that might cause a mal-positioned screw from this percutaneous procedure, a logistic regression analysis was carried. The chosen factors are shown in Table 4. Because of the relatively small number of enrolled patients, a stepwise method of logistic regression test was applied. However, we failed to find a significant risk factor for mal-positioned screw using logistic regression analysis. Using the receiver operating characteristic, the area under the curve in the axial and coronal views was 0.813 and 0.671, respectively, in the ISS group (Fig. 2A), and 0.461 and 0.792 respectively, in the TITS group (Fig. 2B).

**Table 4:** Logistic regression of risk factors for mal-angulation of the ISS and TITS.

	ISS, axial	ISS, coronal	TITS, axial	TITS, coronal
BMI	0.79	0.40	0.19	0.29
AO	0.40	0.23	0.98	0.99
Sacral fracture	0.80	0.66	0.89	0.76
Open/closed reduction	0.34	0.51	0.80	0.15
Supine/prone position	0.15	0.46	0.84	0.94
Reduction quality	0.96	0.16	0.62	0.33
Sacral dysmorphism	N/A	N/A	0.41	0.46

*ISS* iliosacral screw, *TITS* trans-iliac trans-sacral screw, *BMI* body mass index, *AO* Arbeitsgemeinschaft für Osteosynthesefragen, *N/A* not available.

Several univariate analyses were conducted to attempt to find potential risk factors for mal-positioned screws (Table 5). No significant risk factors were found in the ISS group, regardless of mpCT view. Although no risk factor was found in the coronal view for the TITS group, the patient’s body mass index (BMI) was identified as a single significant risk factor (p = 0.02) in the axial view.

**Table 5:** Univariate analysis of risk factors for mal-angulation of the ISS and TITS.

	ISS, axial	ISS, coronal	TITS, axial	TITS, coronal
BMI	0.55	0.99	0.02*	0.87
AO	0.71	0.49	0.28	1
Sacral fracture	1	1	1	0.47
Open/closed reduction	0.48	0.53	1	0.34
Supine/prone position	1	0.52	1	0.68
Reduction quality	0.94	0.21	0.46	0.53
Sacral dysmorphism	N/A	N/A	0.22	0.20

*ISS* iliosacral screw, *TITS* trans-iliac trans-sacral screw, *BMI* body mass index, *AO* Arbeitsgemeinschaft für Osteosynthesefragen, *N/A* not available.

*p < 0.05.

## Discussion

In this study, we reviewed the efficacy and safety of percutaneous ISS and TITS insertion using a single-arm fluoroscopic intensifier intraoperatively in 85 patients with pelvic ring injuries. The results revealed a satisfactory fracture reduction quality and a low rate of mal-positioned screws. Although BMI was a potential risk factor of screw mal-positioning according to the Mann–Whitney U test, it failed to show significance in logistic regression analysis.

The application of ISS and TITS has been widely accepted for the treatment of posterior pelvic ring injuries^26–28^. However, mal-positioned screws can cause devastating consequences, such as injuries to superior gluteal vessels, iliac vessels, lumbosacral nerve roots, and sympathetic chain^4,9,29–31^. Therefore, for effective and safe osteosynthesis, it is critical to perform this procedure under intraoperative real-time image guidance.

The optimal corridors of the ISS and TITS are narrow, and the procedure requires a high degree of technical skill. Therefore, several intraoperative image assessment technologies have been applied to enhance the accuracy of screw placement. Peng et al. compared the one and two C-arm fluoroscope technique, finding similar mal-positioned and clinical complication rates, but the two C-arm group had a shorter operation time (16 vs. 45 min, p < 0.001) and lower radiation exposure (4.5 vs. 5.7 min, p < 0.001)^16^. A recent study by Ciolli et al., showed satisfactory accuracy using the O-arm, with a complication rate and mal-positioning of screw varying from 0 to 15%^17^. Berger-Groch et al. compared fluoroscope-based conventional technique with 2D navigation procedures and found similar rates of malposition, but the radiation dose using the conventional technique was twice that of the 2D navigation procedure^32^. Richter et al. found that intraoperative computed tomography significantly reduced screw perforation rate compared to that when using conventional 3-dimensional navigation^18^. Although the advantages of advanced image systems cannot be ignored, the clinical utility might be limited to a few medical institutes. Because the single-arm fluoroscopic intensifier is the most common image assessment tool in most facilities, preoperative preparation is critical; personal protections from radiational exposure, such as keeping surgeon’s hand out of the field, covering thyroid and body with lead cloths, wearing lead spectacles, laser guidance, and radiation awareness, are crucial for medical staff in the operation theater^33,34^.

The reduction quality of the pelvic ring injuries in this study were comparable to those of previous reports^35–38^, which could be due to the treatment sequence. Before implanting the screws percutaneously, all the fractures should be reduced as possible. For AO B2.1 injuries, the reduction of the pelvic ring was initiated from the anterior pelvic ring to externally rotate the affected hemipelvis. A similar reduction sequence was indicated for AO B3.1 and B3.2 injuries. A considerable percentage (70.8%) of the patients underwent open reduction for diastatic sacroiliac joints because as long as anatomical reduction was achieved, a well-positioned screw could be inserted despite a prone position takes additional time to correct positioning of the patients and is more bothersome to anesthesiologists^39^. Patients with vertically unstable sacral fractures underwent a cranial-caudal orientation reduction prior to osteosynthesis. Using this “reduction first” concept, we achieved a low malposition rate, with no implantation-related complications.

A high BMI may limit the application of percutaneous surgeries^40–42^. We identified that a higher BMI was the single risk factor of TITS screw angle error under axial view of computed tomography (*p* = 0.02). This finding may result from the cumbersome patient positioning, blurry fluoroscopic images, or difficulty in instrument application for obese patients. However, it failed to reveal its significance during logistic regression analysis. We postulated that the reasons of inconsistence between two statistical results were due to our relatively small case number and the low malposition rate (4.8%) of the percutaneously applied screws.

Sacral dysmorphism is defined as upper sacral segment dysplasia and have a higher risk in mal-positioned implant during percutaneously placing ISS and TITS^43^. In dysmorphic sacrum, narrow but adequate corridor for ISS at S1 segment can be found; however, it carries a considerable rate of malalignment^43,44^. Currently, it is believed that the use of a 3D navigation system during operation confers a lower rate of mal-positioned screw^44,45^. The incidence of sacral dysmorphism in our cohort was 6.6%. All dysmorphic sacrum underwent percutaneous TITS osteosynthesis, and there were no complications that required revised surgeries under single C-arm fluoroscope intensifier examination. Additionally, the morphology variation of the sacrum was not a factor in mal-angulation of the screws. A similar concept reported by Rommens et al.^43^ showed that using 2D-fluoroscopic-guided ISS osteosynthesis would be a safe procedure in clinical practice if a thorough preoperative evaluation of the morphology of the upper sacrum, recognition of all the necessary anatomical landmarks, and careful operative procedure were performed.

This study has several limitations. First, its retrospective design study has inherited limitations. The cohort was treated using a single intraoperative image evaluation without a comparative method such as navigation-assisted percutaneous screw osteosynthesis. Second, only a small number of patients were enrolled. Third, the treatment protocol might have some divergence for patients with a similar fracture type such as supine or prone position and closed or open reduction. Fourth, all the operations were performed by a single surgeon, whose experience might have affected the surgical outcomes. However, we found that the radiation dose exposure and reduction quality of the pelvis was acceptable, and no screws penetrated the neuroforamen.

## Conclusions

With careful preoperative planning and intraoperative preparations, percutaneous ISS and TITS implantation under a single-arm fluoroscopic intensifier examination is reliable and safe. Further prospective studies applying different intraoperative image systems should be conducted to identify their potential advantages over this surgical technique.
